# Age at Menarche and Its Association with the Metabolic Syndrome and Its Components: Results from the KORA F4 Study

**DOI:** 10.1371/journal.pone.0026076

**Published:** 2011-10-18

**Authors:** Doris Stöckl, Christa Meisinger, Annette Peters, Barbara Thorand, Cornelia Huth, Margit Heier, Wolfgang Rathmann, Bernd Kowall, Heidi Stöckl, Angela Döring

**Affiliations:** 1 Helmholtz Zentrum München, Institute of Epidemiology II, German Research Center for Environmental Health, Neuherberg, Germany; 2 Department of Obstetrics and Gynaecology, Campus Grosshadern, Ludwig-Maximilians-University, Munich, Germany; 3 Central Hospital of Augsburg, MONICA/KORA Myocardial Infarction Registry, Augsburg, Germany; 4 Institute of Biometrics and Epidemiology, German Diabetes Center, Leibniz Center for Diabetes Research at Heinrich Heine University, Düsseldorf, Germany; 5 London School of Hygiene and Tropical Medicine, Social and Mathematical Epidemiology Group, London, United Kingdom; 6 Helmholtz Zentrum München, Institute of Epidemiology I, German Research Center for Environmental Health, Neuherberg, Germany; Universidad Peruana Cayetano Heredia, Peru

## Abstract

**Objective:**

The metabolic syndrome is a major public health challenge and identifies persons at risk for diabetes and cardiovascular disease. The aim of this study was to examine the association between age at menarche and the metabolic syndrome (IDF and NCEP ATP III classification) and its components.

**Design:**

1536 women aged 32 to 81 years of the German population based KORA F4 study were investigated. Data was collected by standardized interviews, physical examinations, and whole blood and serum measurements.

**Results:**

Young age at menarche was significantly associated with elevated body mass index (BMI), greater waist circumference, higher fasting glucose levels, and 2 hour glucose (oral glucose tolerance test), even after adjusting for the difference between current BMI and BMI at age 25. The significant effect on elevated triglycerides and systolic blood pressure was attenuated after adjustment for the BMI change. Age at menarche was inversely associated with the metabolic syndrome adjusting for age (p-values: <0.001 IDF, 0.003 NCEP classification) and additional potential confounders including lifestyle and reproductive history factors (p-values: 0.001, 0.005). Associations remain significant when additionally controlling for recollected BMI at age 25 (p-values: 0.008, 0.033) or the BMI change since age 25 (p-values: 0.005, 0.022).

**Conclusion:**

Young age at menarche might play a role in the development of the metabolic syndrome. This association is only partially mediated by weight gain and increased BMI. A history of early menarche may help to identify women at risk for the metabolic syndrome.

## Introduction

Menarche, the onset of menstruation in girls, indicates the attainment of reproductive capacity. Age at menarche is inversely associated with adiposity in childhood and adulthood [1]–[5], risk for cardiovascular morbidity and mortality [6], [7] as well as type 2 diabetes mellitus [8], [9]. Efforts to identify women at risk of these diseases earlier in life are very important in order to enable benefit from preventive interventions. Fulfilling the criteria of the metabolic syndrome, a clustering of metabolic risk factors, increases the risk of developing cardiovascular disease and diabetes. The metabolic syndrome has become one of the major public health challenges worldwide [10]. For example, among women in Southern Germany, aged 55 to 74 years, 24 and 46% were categorized as having the metabolic syndrome based on the 2005 National Cholesterol Education Program, Adult Treatment Panel III (NCEP ATP III) and the 2005 International Diabetes Federation (IDF) criteria, respectively [11]. The metabolic syndrome, its single components and its clustering are markers for high health care utilization and high costs among patients receiving medical care [12].

There are different classifications of the metabolic syndrome leading to confusion and an incomparability of studies [10]. However, all definitions are to some degree suitable for the early detection of type 2 diabetes or cardiovascular diseases [13], [14]. Boronat et al. showed that the IDF definition identifies a surplus of individuals whose cardiovascular risk profile, particularly regarding some non-traditional cardiovascular risk factors, is less adverse than that observed in subjects also diagnosed by the NCEP ATP III definition [15].

Few studies have been conducted to examine the association between age at menarche and risk of metabolic syndrome and the findings have been inconclusive [16]–[19]. In addition, most of the published studies on this issue have been conducted in Asian populations, while studies based on European women are still missing.

The aim of this study was to determine whether age at menarche is independently associated with the metabolic syndrome, or whether it is triggered by body mass index (BMI) and other factors. To be better able to compare the results with other studies, we used two main classifications for the metabolic syndrome, the IDF and NCEP ATP III classifications.

## Methods

### Subjects

The KORA F4 study is a follow-up study of the KORA S4 study, a “population-based” health survey conducted in the city of Augsburg and two surrounding counties between 1999 and 2001. A total sample of 6640 subjects was drawn from the target population consisting of all German residents of the region aged 25 to 74 years. The study design, sampling method and data collection have been described in detail elsewhere [20].

Of all 4261 participants of the S4 baseline study, 3080 also participated in the 7-year follow-up F4 study. Persons were considered ineligible for F4 if they had died in the meantime (n = 176, 4%), lived outside the study region, were lost to follow-up (n = 206, 5%), or had demanded deletion of their address data (n = 12, 0.2%). Of the remaining 3867 eligible persons, 174 could not be contacted, 218 were unable to participate in the study because they were too ill or had no time, and 395 were not willing to participate in this follow-up, resulting in a response rate of 79.6%. The current study was restricted to 1594 female subjects, aged 32 to 81 years at follow-up.

We excluded all women for whom no or only incomplete information of age at menarche (n = 35), status of metabolic syndrome (NCEP ATP III and IDF classification) (n = 14) and any of the covariables was available (n = 9). The final analysis therefore included 1536 study participants, after excluding 2 probands due to age at menarche, as described below.

### Ethics Statement

All study participants gave written informed consent and the study was approved by the Ethics Committee of the Bavarian Medical Association.

### Data Collection

Information on sociodemographic variables, physical activity level, medication use, alcohol consumption, smoking habits and reproductive history was obtained by trained medical staff during a standardized face-to-face interview. In addition, all study participants underwent a standardized medical examination that included the collection of fasting venous blood samples and the performance of an oral glucose tolerance test (oGTT) in all subjects without previously known diabetes. All measurement procedures are described in detail elsewhere [20]. Anthropometric measurements were taken after the participants had removed their shoes, heavy clothing and belts. Body height was measured to the nearest 0.1 cm and weight to the nearest 0.1 kg. BMI was calculated as weight [kg] divided by height^2^ [m^2^]. Waist circumference (WC) was measured at the level midway between the lower rib margin and the iliac crest. Recollected BMI was calculated as weight at age 25 years, assessed in the standardized interview [kg] divided by current height^2^ [m^2^] Actual hypertension was defined as use of antihypertensive medication, being aware of having hypertension or blood pressure values greater than 140/90 mmHg. Participants who were engaged more than one hour per week in physical activity in summer or winter were defined as physically active. Known diabetes mellitus was defined as use of antidiabetic medication or self-report which was validated by questioning the treating physician.

### Clinical chemical measurements

A fasting (at least 8 hours) venous blood sample was obtained from all study participants while sitting. Blood glucose was analyzed using a hexokinase method (GLU Flex of Dade Behring, Germany).

Analyses for high-density lipoprotein (HDL) cholesterol were carried out with AHDL Flex of Dade Behring, and fasting triglycerides (TG) were measured with the TGL Flex GPO-PAP assay of Dade Behring.

All additional analyses were carried out using the analyzer Dimension RxL (Dade Behring, Germany).

### Assessment of age at menarche

Age at menarche was defined as age at the first menstrual bleeding, assessed in full years. This information was obtained by a personal interview in the KORA S4 survey. The question was open-ended: “At what age did you have your first menstrual period (menarche)?” We excluded participants with menarche after 18 years (n = 2), because this may likely be due to a pathological status or a recall error. For analysis, age at menarche was categorized into three categories (<12 years, 12–15 years and >15 years).

### Classification of the metabolic syndrome

All subjects were classified whether or not they have the metabolic syndrome according to the current IDF as well as NCEP ATP III criteria. The IDF definition consists for European women of central obesity (≥80 cm WC), plus two of the following symptoms: raised fasting TG (>150 mg/dl or drug treatment for high TG), reduced HDL-cholesterol (<50 mg/dl or drug treatment for this lipid abnormality), raised blood pressure (≥130/85 mmHg or treatment for previously diagnosed hypertension) and raised fasting plasma glucose (≥100 mg/dl or previously diagnosed type two diabetes) [10]. The NCEP ATP III metabolic syndrome classification for women requires fulfillment of at least three of the following criteria: raised fasting plasma glucose (≥100 mg/dl or previously diagnosed type two diabetes), reduced HDL-cholesterol (<50 mg/dl or drug treatment for low HDL-cholesterol), elevated fasting TG (>150 mg/dl or drug treatment for high TG), raised blood pressure (≥130/85 mmHg or treatment for previously diagnosed hypertension) or central obesity (WC≥88 cm) [21].

### Statistical analyses

Basic characteristics of the study population were analyzed stratified by age at menarche (<12 years, 12–15 years and >15 years). For normally distributed variables the mean and standard deviation, for non-normally distributed variables the median and the interquartile range and for categorized variables the percentages were calculated. The p-values are shown for the difference of these variables between the three categories.

Linear regression analyses were fitted with age at menarche supplied both as a categorized variable and in separate analyses as a linear variable and the cardiovascular risk factors as linear dependent variables. Age at menarche between 12 and 15 years was the reference group in the categorized model. Results are presented as least square means (lsmean) with 95% confidence intervals (95% CI).

Fasting TG, HDL, FG and glucose 2 hours after oGTT (2-h glucose) were not normally distributed. Thus, the log-transformed normally distributed variables were used in all analyses. The results are shown as the geometric means after back-transformation.

For the regression analyses several models were fitted, controlling for the following potentially confounding variables. Sociodemographic variables: age (years), education level (8–10, 11–12, 13–17 years), marital status (single, married, divorced or widowed); lifestyle variables: current smoking (yes/no), alcohol intake ≥20 g/day (yes/no) and physical activity regularly more than one hour per week (yes/no); reproductive confounding variables: parity (no pregnancy, 1–2, ≥3 pregnancies), current or ever use of hormone replacement therapy (yes/no), current or ever use of oral or other systemic functioning contraceptives (yes/no); medical confounders: BMI (kg/m^2^), WC (cm), recollected BMI at age 25 (kg/m^2^), BMI change since age 25 (kg/m^2^), HDL-cholesterol (mg/dl), TG (mg/dl), systolic and diastolic blood pressure (mmHg), actual hypertension (yes/no), fasting glucose (FG) and 2–hour glucose (mg/dl) if an oGTT has been conducted. Trend tests were performed by using age at menarche as a linear variable.

Additionally, a test of interaction has been conducted for age at examination with age at menarche (in years) for all analyzed variables. The tests showed no statistically significant interaction (all p-values≥0.05).

Logistic regression models were performed to test the association between categorized age at menarche and the metabolic syndrome (both definitions). Confounder adjustment was done as described for the linear regression models.

Significance tests were two-tailed and p-values less than 0.05 were considered statistically significant. All analyses were performed using SAS (version 9.1, SAS Institute Inc, Cary, NC, USA).

## Results

The mean age at menarche in our study sample was 13.5 years, with a standard deviation of 1.6. The mean age of the study participants was 55.6 years with a standard deviation of 13.1. The sample characteristics by age at menarche are provided in Table 1. Age at time of survey shows the strongest relationship with age at menarche. In summary, women reporting an age at menarche below 12 years had a lower current age, a higher mean BMI, a greater WC and a higher recollected BMI at age 25 years, were more likely to be a current smoker and less physically active than women with an age at menarche between 12 and 15 years. Furthermore, women with an age at menarche below 12 years more often suffered from diabetes mellitus, had higher mean TG, lower mean HDL-cholesterol and higher FG levels in comparison to women with an age at menarche between 12 and 15 years. Women with an age at menarche above 15 years were older, less likely to smoke, more likely to be physically active and to be divorced than women with an age at menarche between 12 and 15 years. In addition, they had less often ever used oral contraceptives and were more likely to have actual hypertension.

**Table 1:** Sample characteristics, by age at menarche.

Age at menarche (years)	<12			12–15			>15			
	n = 121			n = 1257			n = 158			
	%	Mean	SD	%	Mean	SD	%	Mean	SD	p-value
Age at time of the survey (years)		53.1	12.0		55.1	12.9		61.5	13.1	<0.001
Body mass index (kg/m^2^)		28.2	5.6		27.3	5.3		27.0	5.0	0.136
Waist circumference (cm)		89.7	13.9		88.0	13.3		87.8	12.7	0.369
Recalled BMI at age 25 (kg/m^2^)		23.4	4.4		22.3	3.3		22.4	3.4	0.004
HDL- cholesterin^2^ (mg/dl)		58.4	13.1		61.6	14.4		61.0	13.9	0.063
Fasting triglycerides^2^ (mg/dl)		123.4	88.0		105.1	64.6		114.9	62.6	0.005
Alcohol consumption (>20 g/day)	19.8			15.1			10.8			0.231
Current smoker (%)	19.8			15.4			12.7			0.257
Systolic blood pressure (mmHg)		117.7	17.6		116.9	18.0		117.7	18.9	0.809
Diastolic blood pressure (mmHg)		74.0	8.9		72.8	9.3		72.5	10.2	0.388
Diabetes mellitus (%)	9.1			5.5			7.6			0.187
Actual hypertension^3^ (%)	33.9			32.5			38.6			0.248
Physically active (more than one hour per week) (%)	41.3			56.6			58.9			0.004
Pregnancies (%)										0.205
0 pregnancies	17.4			14.2			17.7			
1–2 pregnancies	48.8			50.0			40.5			
≥3 pregnancies	33.9			35.9			41.8			
Education (years) (%)										0.128
8–10	47.1			48.3			58.2			
11–12	25.6			22.9			21.5			
13–17	27.3			28.8			20.3			
Marital status (%)										0.149
Single	14.0			9.1			9.5			
Married	70.2			69.8			64.9			
Divorced or widowed	15.7			21.1			25.9			
Current use of systemic hormone replacement therapy (%)	9.9			8.7			10.8			0.653
Ever use of systemic hormone replacement therapy (%)	32.2			29.4			31.6			0.699
Current use of oral/systemic contraceptives (%)	8.3			6.1			7.0			0.623
Ever use of oral/systemic contraceptives (%)	74.4			74.8			65.8			0.054
Fasting glucose^2^ (mg/dl)		97.6	21.4		94.0	14.9		96.0	17.9	0.029
2-h glucose^2^ (mg/dl)		105.0	43.0		103.0	39.0		104.0	40.0	0.296
HbA1c (%)		5.5	0.5		5.5	0.5		5.6	0.6	0.016

Abbreviation: SD: Standard deviation.

p-value, testing the difference between the 3 categories of age at menarche.

2 the median and the interquartile range are given due to a non-normal distribution of these variables.

3 defined as use of antihypertensive medication, being aware of having hypertension or blood pressure values greater than 140/90 mmHg.

The association between age at menarche in three categories and single components of the metabolic syndrome and cardiovascular risk factors is shown in Table 2 and in Table S1. Additional analysis of age at menarche as linear variable confirmed the results of the analyses with the categorized variable. Both analyses showed an inverse association between age at menarche and BMI, WC, FG and 2-h glucose. The relationship with systolic blood pressure and TG was attenuated after adjustment for recollected BMI at age 25 or the BMI change since age 25 years. There was no statistically significant association between age at menarche and HDL cholesterol levels as well as diastolic blood pressure.

**Table 2:** Age at menarche and its association with selected cardiovascular risk factors and components of the metabolic syndrome.

Age at menarche	<12			12–15			>15			
(years)	n = 121			n = 1257			n = 158			
	lsmean	CI		lsmean	CI		lsmean	CI		p-value for trend*
**BMI (kg/m^2^)**										
Age-adjusted	28.5	27.6	29.4	27.3	27.0	27.6	26.3	25.5	27.1	<0.001
Complete model	27.9	27.0	28.8	26.9	26.4	27.4	25.9	25.0	26.8	<0.001
Complete+BMI	28.2	27.6	28.9	27.2	26.9	27.5	27.1	26.5	27.7	<0.001
**Waist circumference (cm)**										
Age-adjusted	90.6	88.4	92.8	88.2	87.5	88.9	85.5	83.6	87.5	<0.001
Complete model	89.8	87.5	92.1	87.8	86.7	89.0	85.3	83.2	87.5	<0.001
Complete+BMI	90.6	88.9	92.3	88.6	87.7	89.3	88.1	86.5	89.7	0.002
**Systolic blood pressure (mmHg)**										
Age-adjusted	119.1	116.2	122.1	117.2	116.3	118.1	114.4	111.8	117.0	0.017
Complete model	118.3	115.1	121.4	116.6	115.1	118.2	113.9	111.0	116.8	0.026
Complete+BMI	118.3	115.2	121.4	116.5	115.0	118.0	114.6	111.7	117.5	0.049
**Diastolic blood pressure (mmHg)**										
Age-adjusted	74.1	72.4	75.7	72.9	72.3	73.4	72.3	70.8	73.8	0.268
Complete model	73.6	71.8	75.4	72.5	71.6	73.3	72.0	70.3	73.6	0.321
Complete+BMI	73.7	71.9	75.4	72.5	71.7	73.4	72.5	70.8	74.1	0.485
**Fasting triglycerides (mg/dl)**										
Age-adjusted	107.7	98.8	117.4	92.3	89.9	94.8	92.8	86.0	100.1	0.015
Complete model	107.5	98.1	117.7	93.1	89.1	97.4	93.9	86.3	102.2	0.021
Complete+BMI	108.8	99.7	118.7	94.3	90.3	98.4	97.6	89.9	105.9	0.060
**HDL cholesterol (mg/dl)**										
Age-adjusted	57.0	54.6	59.4	59.9	59.2	60.7	59.5	57.4	61.8	0.123
Complete model	57.1	54.7	59.6	59.6	58.3	60.9	59.1	56.8	61.5	0.152
Complete+BMI	56.6	54.4	59.0	59.1	58.0	60.3	57.8	55.6	60.1	0.351
**Fasting glucose (mg/dl)**										
Age-adjusted	96.9	94.7	99.3	93.3	92.6	93.9	92.4	90.5	94.3	<0.001
Complete model	96.5	94.1	98.9	93.0	91.9	94.2	92.1	90.0	94.3	<0.001
Complete+BMI	96.7	94.5	99.0	93.4	92.3	94.5	93.6	91.5	95.6	0.003
**2-h glucose** ** **(mg/dl)**										
Age-adjusted	110.6	104.8	116.7	104.9	103.2	106.6	101.9	97.1	106.9	0.003
Complete model	108.9	102.9	115.2	103.7	100.9	106.6	100.7	95.5	106.1	0.004
Complete+BMI	109.6	103.9	115.7	104.3	101.6	107.1	102.8	97.6	108.2	0.009

Abbreviations: CI: Confidence Interval, BMI: Body mass index, HDL: High density lipoprotein, lsmean: least square means.

*the p-values for trend are reported for age at menarche as continuous variable.

**2-h glucose: glucose 2 hours after an oral glucose challenge; n = 1417 due to the non-performance of the oGTT in people with diagnosed diabetes.

Model 1: results were adjusted for age (in years).

Model 2: “complete model” results were adjusted for age (in years), number of pregnancies (no pregnancy, 1–2 pregnancies, more than 2 pregnancies), ever use of oral contraceptives, ever use of hormone replacement therapy, physical activity (less and more than one hour per week), smoking habits (current or no current smoking) and alcohol intake (more than 20 g per day).

Model 3: “complete plus BMI change” results were adjusted for all variables in model 2 plus the BMI change since age 25 years.

Age at menarche showed no association with education level, marital status, current use of hormone replacement or oral contraceptive use and these variables were therefore not included in the models.

There were 421 cases with the metabolic syndrome using the IDF and 383 cases using the NCEP ATP III classification.

Early age at menarche was significantly associated with the metabolic syndrome after controlling for age (p-values for trend with age at menarche as continuous variable: <0.001 IDF, 0.003 NCEP ATP III) (Table 3). Adjustment for age, physical activity, smoking habits, alcohol consumption, parity, ever use of oral contraceptives or ever use of hormone replacement therapy showed practically unchanged p-values. A model including recollected BMI at age 25 years to the lifestyle and reproductive adjusted model showed a weaker association with p-values of 0.008 and 0.033. The model with adjustement for the BMI change since age 25 years in addition to all other confounding factors is comparable to the results of the model with all confounders additionally to the recollected BMI at age 25 years (p-values: 0.005 IDF, 0.022 NCEP) (Table 3). Later age at menarche showed no significant associations with the metabolic syndrome in comparison to the age at menarche from 12 to 15 years, but a trend towards lower odds ratios was seen.

**Table 3:** Odds ratios for metabolic syndrome (IDF and NCEP ATP III Classification) according to age at menarche.

Age at menarche	<12			12–15	>15			
(years)				(reference)				
	OR	CI		OR	OR	CI		p-value for trend*
**Classification IDF**								
Number of cases: 421								
Model 1	1.65	1.07	2.55	1.00	0.79	0.54	1.17	<0.001
Model 2	1.54	0.99	2.38	1.00	0.80	0.54	1.20	0.001
Model 3	1.66	1.07	2.56	1.00	0.79	0.53	1.17	<0.001
Model 4	1.55	1.00	2.42	1.00	0.80	0.53	1.19	0.001
Model 5	1.43	0.91	2.25	1.00	0.79	0.52	1.20	0.008
Model 6	1.70	1.06	2.72	1.00	0.97	0.62	1.51	0.005
**Classification NCEP ATP III 2005**								
Number of cases: 383								
Model 1	1.69	1.08	2.64	1.00	0.83	0.56	1.24	0.003
Model 2	1.57	1.00	2.47	1.00	0.84	0.56	1.27	0.006
Model 3	1.69	1.08	2.65	1.00	0.83	0.56	1.24	0.003
Model 4	1.58	1.00	2.49	1.00	0.84	0.56	1.26	0.005
Model 5	1.45	0.91	2.30	1.00	0.86	0.56	1.31	0.033
Model 6	1.79	1.09	2.92	1.00	1.08	0.68	1.71	0.022

*p-values for trend with age at menarche as linear variable.

Abbreviations: OR: odds ratio, CI: confidence interval.

Model 1: results were adjusted for age (in years).

Model 2: “Life-style model”: results were adjusted for age (in years), physical activity (less or more than one hour per week), current smoking (yes/no) and alcohol intake (less or equal and more than 20 g per day).

Model 3: “reproductive model”: results were adjusted for age (in years), number of pregnancies (no pregnancy, 1–2 pregnancies, more than 2 pregnancies), ever use of oral contraceptives and ever use of hormone replacement therapy.

Model 4: “complete model”: results were adjusted for all above mentioned factors (model 2 and 3).

Model 5: results were adjusted for all the variables in model 4 plus recollected BMI at age 25 years.

Model 6: results were adjusted for all the variables in model 4 plus the BMI change since age 25 years.

## Discussion

This study shows that low age at menarche is associated with a higher risk of having the metabolic syndrome and most of its single components in women aged 32 to 81 years, even after controlling for potential confounders (age, lifestyle factors, reproductive history, recollected BMI at age 25 years, and BMI change since age 25 years). Early onset of menarche in comparison to the group age at menarche between 12 and 15 years was associated with the metabolic syndrome defined according to the NCEP ATP III and the IDF classification. Later age at menarche showed no significant association but a trend towards lower odds ratios was seen. The results are quite similar for the two different definitions of the metabolic syndrome.

Our study is in good agreement with previous studies (1–5) showing that age at menarche is inversely associated with body mass index and waist circumference.

### Results of other studies on the metabolic syndrome and its components

Studies on the association between age at menarche and metabolic syndrome are sparse and present contradictory results [16]–[19].

A study of 892 postmenopausal women who participated in the 2005 Korean National Health and Nutrition Survey showed no association between age at menarche and metabolic syndrome [16]. The Bogalusa Heart Study conducted among 1479 young adult women (19–37 years), found that early menarche is characterized by a higher prevalence of clustered risk variables, which are part of the metabolic syndrome. Unfortunately, this study did not examine the effect directly on the metabolic syndrome [18]. A further study including 7349 Chinese women aged 50 to 92 years showed an odds ratio of 1.49 (CI 1.22–1.82) for the metabolic syndrome in women with an age at menarche below 12.5 years, using the NCEP ATP III classification [19]. A study of 9000 Chinese women aged 25 to 64 years reported that age at menarche was inversely associated with the total number of metabolic syndrome components and the metabolic syndrome using the IDF classification [17].

A previous literature review, published in 1999 on reproductive history and cardiovascular disease risk in postmenopausal women showed no association between age at menarche and cardiovascular risk factors [22]. However, recent publications don't support this result. The Epic Norfolk cohort study showed in 15,807 participants that age at menarche below 12 years is associated with incident cardiovascular disease (1.17; 1.07–1.27), incident coronary heart disease (1.23; 1.06–1.62) and a higher risk of hypertension (1.13; 1.02–1.24). These associations were only partly mediated by adiposity [6]. Additionally, a study of 794 Finnish women found that early menarche is only a risk marker, and that greater childhood BMI contributes to the earlier age at menarche and hence increases adult BMI and subsequently cardiovascular risk [7]. On the contrary, a Japanese cohort study with 37,965 postmenopausal women aged 40–79 years showed no association between age at menarche and mortality of coronary heart disease [23]. In our study, we examined the association of age at menarche and its association with individual components of the metabolic syndrome, which represent selected cardiovascular risk factors, e.g. systolic blood pressure or TG which showed a significant association before adjustment for the BMI at age 25 or the BMI change since.

Gunderson et al. reported that childbearing is associated with higher incidence of the metabolic syndrome among women of reproductive age controlling for measurements of the components of the metabolic syndrome before pregnancy [24]. A study with Chinese women confirmed these results [25]. Other studies showed that this effect is a combination of lifestyle risk factors and/or biological changes associated with childbearing, which may explain the positive association between parity and increased risk of metabolic syndrome [16], [26]. In contrast to these studies, Cho et.al. found, that age at first birth was negatively associated with the metabolic syndrome, but not parity in 892 postmenopausal women [16]. We did not see an association between parity and the presence of the metabolic syndrome, which is probably due to lack of power in our study.

The role of menarche in the development of the metabolic syndrome is not yet clear. It is possible that early menarche only presents a marker for childhood obesity. Whether it acts additionally or as a risk factor by itself or through sex hormone differences over the life span needs to be assessed. Existing data suggest that about half of the variance in the timing of menarche is due to genetic factors [27], and there seems to be a genetic basis for the phenotypic associations between age at menarche and BMI [28], [29]. In addition, it was found that obesity in childhood is associated with metabolic syndrome in adolescence and adulthood [7], [30]. The importance of targeting girls for anti obesity interventions has therefore been emphasized [5], [31]. Large prospective studies are needed to understand the underlying mechanisms by which menarche increases the risk for metabolic syndrome and risk factors for cardiovascular diseases in adulthood.

### Strengths and limitations

The strength of the current study is its representativeness since results are based on a large sample, drawn from the general population. Another strong point is the availability of data on lifestyle, reproductive history and multiple cardiovascular risk factors, measured according to a standardized protocol. Compared to other studies, weight and height data are based on measurements rather than self-reported data, which reduces biases as participants tend to under-report weight and over-report height [32]. Another great advantage is the wide age-range in our study (between 32 and 81 years) since other studies only show results either for postmenopausal female subjects or for subjects in younger adulthood.

Several limitations should be mentioned. Misclassification of age at menarche might have happened due to the retrospective assessment of this variable. However, studies have shown that the actual reported mean age at menarche did not differ from the mean age at menarche recalled 33 years later [33], [34]. In addition, misclassification of age at menarche is not likely to be associated with the metabolic syndrome. This study unfortunately could not adjust for premenarcheal BMI, which we replaced with the variable recollected body weight at age 25. It is not possible to obtain information on childhood BMI for our population based sample of women, because registers or child health care centers where this data could be recorded don't exist in Germany. It has been shown that the BMI in the twenties is strongly correlated with the BMI in the youth and in the teenage years [35]. Nevertheless, recalls of BMI at age 25 are still subject to recall biases and don't rule out additional confounding. Misclassification of other variables needs to be considered since some of the covariables were measured by self reports of the subjects, like alcohol consumption, smoking habits and physical activity. A further limitation of this study is that it has a cross-sectional design, implicating that cause and effect relationships cannot be discerned.

### Conclusion and implication

In conclusion, the present study showed that an earlier age at menarche plays a role in the development of the metabolic syndrome. This association is only partially mediated by weight gain and increased BMI. A history of early menarche, especially before the age of 12 years, may help to identify women at risk for the metabolic syndrome and an increased body mass index. Early identification of women at risk may help to prevent metabolic syndrome and the subsequent development of cardiovascular diseases and diabetes.

## Supporting Information

Table S1 **Age at menarche and its association with selected cardiovascular risk factors and components of the metabolic syndrome.**
(DOC)
