# Supplementary material for: Age at Menarche and Its Association with the Metabolic Syndrome and Its Components: Results from the KORA F4 Study
Source: PLoS One. 2011 Oct 18;6(10):e26076. doi: 10.1371/journal.pone.0026076 (PMC3196515; doi:10.1371/journal.pone.0026076)
Supplement: Table S1 — Age at menarche and its association with selected cardiovascular risk factors and components of the metabolic syndrome. (DOC) [file pone.0026076.s001.doc]

Supplement Table: Age at menarche and its association with selected cardiovascular risk factors and components of the metabolic syndrome

| **Age at menarche**  (years) |  | < 12  n = 121 | | |  |  | 12 – 15  n = 1257 | | |  |  | >15  n = 158 | | |  |  |
| --- | --- | --- | --- | --- | --- | --- | --- | --- | --- | --- | --- | --- | --- | --- | --- | --- |
|  | lsmean | | CI | | | lsmean | | CI | | | lsmean | | CI | | | p-value for trend* |
| **BMI** (kg/m²) | | |  |  | |  | |  |  | |  | |  |  | |  |
| Model 1 | 28.5 | | 27.6 | 29.4 | | 27.3 | | 27.0 | 27.6 | | 26.3 | | 25.5 | 27.1 | | <0.001 |
| Model 2 | 27.9 | | 27.0 | 28.9 | | 27.0 | | 26.6 | 27.3 | | 26.0 | | 25.1 | 26.8 | | <0.001 |
| Model 3 | 28.5 | | 27.5 | 29.4 | | 27.2 | | 26.9 | 27.6 | | 26.3 | | 25.4 | 27.1 | | <0.001 |
| Model 4 | 27.9 | | 27.0 | 28.8 | | 26.9 | | 26.4 | 27.4 | | 25.9 | | 25.0 | 26.8 | | <0.001 |
| Model 5 | 27.0 | | 26.3 | 27.8 | | 26.9 | | 26.5 | 27.3 | | 25.9 | | 25.2 | 26.6 | | 0.015 |
| Model 6 | 28.2 | | 27.6 | 28.9 | | 27.2 | | 26.9 | 27.5 | | 27.1 | | 26.5 | 27.7 | | <0.001 |
| **Waist circumference** (cm) | | |  |  | |  | |  |  | |  | |  |  | |  |
| Model 1 | 90.6 | | 88.4 | 92.8 | | 88.2 | | 87.5 | 88.9 | | 85.5 | | 83.6 | 87.5 | | <0.001 |
| Model 2 | 89.9 | | 87.7 | 92.2 | | 88.0 | | 87.1 | 89.0 | | 85.5 | | 83.4 | 87.5 | | <0.001 |
| Model 3 | 90.5 | | 88.3 | 92.8 | | 88.0 | | 87.1 | 88.9 | | 85.4 | | 83.4 | 87.5 | | <0.001 |
| Model 4 | 89.8 | | 87.5 | 92.1 | | 87.8 | | 86.7 | 89.0 | | 85.3 | | 83.2 | 87.5 | | <0.001 |
| Model 5 | 88.1 | | 86.0 | 90.1 | | 87.9 | | 86.9 | 88.9 | | 85.5 | | 83.6 | 87.4 | | 0.054 |
| Model 6 | 90.6 | | 88.9 | 92.3 | | 88.6 | | 87.7 | 89.3 | | 88.1 | | 86.5 | 89.7 | | 0.002 |
| **Systolic blood pressure** (mmHg) | | | |  | |  | |  |  | |  | |  |  | |  |
| Model 1 | 119.1 | | 116.2 | 122.1 | | 117.2 | | 116.3 | 118.1 | | 114.4 | | 111.8 | 117.0 | | 0.017 |
| Model 2 | 118.1 | | 115.0 | 121.2 | | 116.5 | | 115.2 | 117.8 | | 113.7 | | 111.9 | 116.5 | | 0.026 |
| Model 3 | 119.3 | | 116.3 | 122.3 | | 117.4 | | 116.2 | 118.6 | | 114.6 | | 111.9 | 117.3 | | 0.017 |
| Model 4 | 118.3 | | 115.1 | 121.4 | | 116.6 | | 115.1 | 118.2 | | 113.9 | | 111.0 | 116.8 | | 0.026 |
| Model 5 | 117.8 | | 114.6 | 120.9 | | 116.3 | | 114.7 | 117.8 | | 113.6 | | 110.6 | 116.5 | | 0.037 |
| Model 6 | 118.3 | | 115.2 | 121.4 | | 116.5 | | 115.0 | 118.0 | | 114.6 | | 111.7 | 117.5 | | 0.049 |
| **Diastolic blood pressure** (mmHg) | | | | | | | | | | | | | | | | |
| Model 1 | 74.1 | | 72.4 | 75.7 | | 72.9 | | 72.3 | 73.4 | | 72.3 | | 70.8 | 73.8 | | 0.268 |
| Model 2 | 73.8 | | 72.0 | 75.5 | | 72.6 | | 71.9 | 73.4 | | 72.1 | | 70.6 | 73.7 | | 0.317 |
| Model 3 | 74.0 | | 72.3 | 75.7 | | 72.8 | | 72.1 | 73.5 | | 72.2 | | 70.7 | 73.7 | | 0.267 |
| Model 4 | 73.6 | | 71.8 | 75.4 | | 72.5 | | 71.6 | 73.3 | | 72.0 | | 70.3 | 73.6 | | 0.321 |
| Model 5 | 73.3 | | 71.5 | 75.1 | | 72.4 | | 71.5 | 73.2 | | 71.9 | | 70.3 | 73.6 | | 0.459 |
| Model 6 | 73.7 | | 71.9 | 75.4 | | 72.5 | | 71.7 | 73.4 | | 72.5 | | 70.8 | 74.1 | | 0.485 |
| **Fasting triglycerides** (mg/dl) | | |  |  | |  | |  |  | |  | |  |  | |  |
| Model 1 | 107.7 | | 98.8 | 117.4 | | 92.3 | | 89.9 | 94.8 | | 92.8 | | 86.0 | 100.1 | | 0.015 |
| Model 2 | 109.1 | | 99.8 | 119.2 | | 94.8 | | 91.3 | 98.4 | | 95.3 | | 87.9 | 103.3 | | 0.022 |
| Model 3 | 106.4 | | 97.4 | 116.2 | | 91.0 | | 87.8 | 94.3 | | 91.7 | | 84.7 | 99.3 | | 0.014 |
| Model 4 | 107.5 | | 98.1 | 117.7 | | 93.1 | | 89.1 | 97.4 | | 93.9 | | 86.3 | 102.2 | | 0.021 |
| Model 5 | 105.2 | | 96.0 | 115.2 | | 93.1 | | 89.1 | 97.4 | | 93.0 | | 85.4 | 101.2 | | 0.061 |
| Model 6 | 108.8 | | 99.7 | 118.7 | | 94.3 | | 90.3 | 98.4 | | 97.6 | | 89.9 | 105.9 | | 0.060 |
| **Age at menarche**  (years) |  | <12 | | |  |  | 12 - 15 | | |  |  | > 15 | | |  |  |
|  | lsmean | | CI | | | lsmean | | CI | | | lsmean | | CI | | | p-value for trend |
| **HDL cholesterol** (mg/dl) | | |  |  | |  | |  |  | |  | |  |  | |  |
| Model 1 | 57.0 | | 54.6 | 59.4 | | 59.9 | | 59.2 | 60.7 | | 59.5 | | 57.4 | 61.8 | | 0.123 |
| Model 2 | 57.1 | | 54.7 | 59.5 | | 59.5 | | 58.5 | 60.6 | | 59.2 | | 57.0 | 61.5 | | 0.166 |
| Model 3 | 57.1 | | 54.7 | 59.5 | | 60.0 | | 59.0 | 61.1 | | 59.4 | | 57.2 | 61.7 | | 0.117 |
| Model 4 | 57.1 | | 54.7 | 59.6 | | 59.6 | | 58.3 | 60.9 | | 59.1 | | 56.8 | 61.5 | | 0.152 |
| Model 5 | 57.6 | | 55.2 | 60.0 | | 59.4 | | 58.3 | 60.7 | | 59.1 | | 56.8 | 61.5 | | 0.356 |
| Model 6 | 56.6 | | 54.4 | 59.0 | | 59.1 | | 58.0 | 60.3 | | 57.8 | | 55.6 | 60.1 | | 0.351 |
| **Fasting glucose** (mg/dl) | | | |  | |  | |  |  | |  | |  |  | |  |
| Model 1 | 96.9 | | 94.7 | 99.3 | | 93.3 | | 92.6 | 93.9 | | 92.4 | | 90.5 | 94.3 | | <0.001 |
| Model 2 | 96.9 | | 94.5 | 99.3 | | 93.5 | | 92.6 | 94.5 | | 92.7 | | 90.7 | 94.8 | | <0.001 |
| Model 3 | 96.6 | | 94.3 | 99.0 | | 92.9 | | 92.0 | 93.8 | | 91.9 | | 89.9 | 93.9 | | <0.001 |
| Model 4 | 96.5 | | 94.1 | 98.9 | | 93.0 | | 91.9 | 94.2 | | 92.1 | | 90.0 | 94.3 | | <0.001 |
| Model 5 | 96.0 | | 93.6 | 98.4 | | 93.0 | | 91.9 | 94.2 | | 92.2 | | 90.1 | 94.4 | | 0.002 |
| Model 6 | 96.7 | | 94.5 | 99.0 | | 93.4 | | 92.3 | 94.5 | | 93.6 | | 91.5 | 95.6 | | 0.003 |
| Model 7 | 108.5 | | 105.9 | 111.2 | | 105.2 | | 103.5 | 106.9 | | 105.6 | | 103.1 | 108.1 | | 0.026 |
| **2-h glucose**** (mg/dl) | | | | | | | | | | | | | | | | |
| Model 1 | 110.6 | | 104.8 | 116.7 | | 104.9 | | 103.2 | 106.6 | | 101.9 | | 97.1 | 106.9 | | 0.003 |
| Model 2 | 107.7 | | 101.9 | 113.8 | | 102.6 | | 100.3 | 104.9 | | 99.8 | | 94.9 | 105.0 | | 0.004 |
| Model 3 | 111.8 | | 105.8 | 118.1 | | 106.1 | | 103.8 | 108.5 | | 102.9 | | 97.8 | 108.1 | | 0.002 |
| Model 4 | 108.9 | | 102.9 | 115.2 | | 103.7 | | 100.9 | 106.6 | | 100.7 | | 95.5 | 106.1 | | 0.004 |
| Model 5 | 108.0 | | 102.0 | 114.3 | | 103.7 | | 100.9 | 106.6 | | 100.0 | | 94.8 | 105.4 | | 0.004 |
| Model 6 | 109.6 | | 103.9 | 115.7 | | 104.3 | | 101.6 | 107.1 | | 102.8 | | 97.6 | 108.2 | | 0.009 |

Abbreviations: CI: Confidence Interval, BMI: Body mass index, HDL: High density lipoprotein, lsmean: least square means

*the p-values for trend are reported for age at menarche as continuous variable

** 2-h glucose: glucose 2 hours after an oral glucose challenge; n=1417 due to the non-performance of the oGTT with people with diagnosed diabetes

Model 1: results were adjusted for age (in years)

Model 2: “Life-style model”results were adjusted for age (in years), physical activity (less and more than one hour per week), smoking habits (current or no current smoking) and alcohol intake (more than 20 g per day)

Model 3: “reproductive model”: results were adjusted for age (in years), number of pregnancies (no pregnancy, 1-2 pregnancies, more than 2 pregnancies), ever use of oral contraceptives and ever use of hormone replacement therapy

Model 4: “complete model” results were adjusted for all above mentioned factors (model 2 and 3)

Model 5: results were adjusted for all variables in model 4 plus recollected BMI at age 25 years

Model 6: results were adjusted for all variables in model 4 plus the BMI change since age 25 years

Model 7: model used only for fasting glucose: results were adjusted for all variables in model 6 plus current use of antidiabetic medication
